# Understanding the role of street medicine in harm reduction: a case study of Street Medicine St. Louis

**DOI:** 10.1186/s12954-025-01313-w

**Published:** 2025-10-06

**Authors:** Ari Gzesh, Jeremiah S. Truel, Danielle R. Adams, Luke Zabotka, Sara Malone, Nathanial S. Nolan

**Affiliations:** 1 Silberman School of Social Work, Hunter College CUNY, New York, NY USA; 2 School of Medicine, Washington University in St. Louis, St. Louis, MO USA; 3 School of Social Work, College of Health Sciences, University of Missouri, Columbia, MO USA; 4 Department of Surgery, Washington University in St. Louis, St. Louis, MO USA; 5 Division of Infectious Disease, Veterans Affairs St. Louis Health Care, B604, 915 N Grand Blvd, St. Louis, MO 63106 USA; 6 Division of Infectious Diseases, Washington University School of Medicine, St. Louis, MO USA

**Keywords:** Street medicine, Harm reduction, Unhoused populations

## Abstract

**Background:**

Homelessness results from, and exacerbates, various social determinants of health, including poverty, racism, and inadequate healthcare access, which are further compounded by physical and mental health challenges. The street medicine movement seeks to address these disparities by providing direct medical care and harm reduction services to unsheltered homeless in low-barrier settings. The development of trust is critical to providing this form of care. Few studies have sought to understand the factors influencing trust development in street medicine encounters.

**Methods:**

This case study of Street Medicine St. Louis aims to explore the factors that influence trust and how trust development impacts unhoused individuals’ perceptions and utilization of services and education provided by Street Medicine St. Louis. Qualitative semi-structured interviews were conducted with 19 participants who receive harm reduction services from Street Medicine St. Louis. Participants were selected via purposive sampling from shelters, encampments, and street locations to ensure a diversity of experiences.

**Results:**

Findings highlight that trust, built through consistent outreach, respectful interactions, and non-judgmental care, directly shaped participants' perceptions and utilization of harm reduction resources, including sterile syringes, fentanyl test strips, and naloxone. Trust facilitated greater receptivity to education and increased willingness to apply harm reduction practices.

**Conclusion:**

Building trust through consistent, respectful, and person-centered outreach is essential for effective harm reduction interventions. Street medicine programs should prioritize relational approaches to enhance uptake and impact of harm reduction services among unhoused populations.

**Supplementary Information:**

The online version contains supplementary material available at 10.1186/s12954-025-01313-w.

## Introduction

Homelessness arises from a constellation of social determinants of health, including extreme poverty, chronic mental illness, medical comorbidities, substance use disorders, and structural racism. Homelessness is further complicated by physical and mental trauma, stigma, and inadequate systems of care to adequately treat this population [1]. This increasingly visible crisis has garnered widespread media attention during the COVID-19 pandemic and subsequent election cycles, highlighting the vulnerability of unhoused populations and the intensified political disputes over the management of homelessness. Approximately 770,000 individuals experience homelessness in the United States at any given time, which is the highest level since national reporting commenced in 2007 [2, 3]. This approximation is considered to be an underestimate of the actual burden, with a significant portion of individuals now living in unsheltered conditions that are difficult to account for [4].

Emerging evidence underscores the poor health outcomes prevalent amongst unhoused individuals [5]. These include heightened risk for chronic conditions, such as hypertension, diabetes, and cardiovascular diseases, communicable diseases, like tuberculosis, and mental health disorders, including depression, anxiety, and schizophrenia [6, 7]. These conditions are worsened by malnutrition, exposure to extremes of weather, and inadequate access to preventative health services, with unsheltered individuals experiencing homelessness fairing the worst [8, 9].

Despite these health disparities, unsheltered adults have been largely overlooked by the healthcare system and have limited access to primary care and other essential services [9]. In response, the street medicine movement has emerged to mitigate these crises, driven by the necessity to deliver healthcare directly to patients in their own environments [10, 11]. “Street medicine,” a term popularized by Jim Withers, is defined as “the practice of providing medical care to unsheltered people experiencing homelessness in locations like encampments, parks, and under bridges” [12]. Street medicine emphasizes building trust through consistent, nonjudgmental interactions, which have been shown to be foundational in effectively engaging unhoused populations in health services [13]. It has been described as a philosophy of care as much as a healthcare delivery model and entails meeting the needs of multiply marginalized communities in the locations where they reside [14]. Organizations such as the HIV Medical Association have recently endorsed the need for these alternative models of care as critical to ending the HIV epidemic, and the Center for Medicaid and Medicare Services has now supported this model through the creation of a point-of-service code for street medicine encounters [15–17].

Street medicine can offer life-saving disease prevention and management, and can be categorized into three models: (1) direct provision of services to individuals on the streets, encompassing primary healthcare and specialty care, such as management of HIV and behavioral health services; (2) temporary clinical interventions aimed at linking individuals with stationary or mobile ambulatory care facilities; and (3) outreach efforts targeting individuals on the streets to connect them with traditional medical services, particularly for those with complex medical and social needs. Street teams—typically comprising prescribers, nurses, social workers, and peer navigators (many of whom have lived experience)—can provide comprehensive care for acute and chronic conditions, including vaccinations, wound care, and medication for substance use disorders. Some street teams act as temporary bridges to brick-and-mortar clinics, while others aim to serve as more permanent sources of care for patients experiencing chronic homelessness [18].

A core component of many street medicine programs is an emphasis on harm reduction, a movement that seeks to collaborate with patients around their own health goals, which may not include shelter/housing or abstinence from substance use [19, 20]. Harm reduction, particularly in the context of treating and preventing infectious diseases among individuals experiencing homelessness and substance use, involves a pragmatic and compassionate approach focused on minimizing the negative health consequences associated with substance use, rather than emphasizing abstinence as the primary goal [13]. Philosophically, harm reduction recognizes drug use is a complex, multifaceted phenomenon that is influenced by social, historical and economic contexts and seeks to offer non-judgmental, non-coercive support to people who use drugs as a means to affirm their autonomy and dignity [21]. Central to harm reduction strategies are practical interventions that prioritize individuals' immediate health and safety needs, acknowledging the complex social determinants that contribute to their circumstances. In substance use treatment, harm reduction is operationalized through low-barrier access to safer use supplies, overdose reversal therapies (i.e., Naloxone), and access to pharmaceutic treatments for substance use disorders, which have been demonstrated to be evidenced-based interventions [22–25].

## Community context

This paper focuses on a street medicine initiative founded by Infectious Disease clinicians in St. Louis, Missouri. St. Louis has been profoundly impacted by the U.S. substance use epidemic and has been navigating the consequences of restrictive laws around the provision of harm reduction services and a vastly underfunded public health infrastructure [26, 27]. Unique to this region is the divorce between St. Louis City and St. Louis County and the bistate spread of the metro area, where patients have different access to social services based on zip code and state of residence [28]. Likewise, the state’s history of racism and segregation has resulted in well-characterized disparities across races and zip codes, extending into the unhoused population where Black individuals are four times as likely to be unsheltered as their White counterparts [29, 30].

The 2023 point-in-time count noted that 6,918 people were experiencing homelessness in Missouri with only 4,068 shelter beds available. Only 21 out of 115 Missouri counties have emergency shelters and this deficit of shelter beds has contributed to the increased percentage of unsheltered individuals, which nearly doubled from 2022 to 2023 [31, 32]. Unfortunately, in line with the recent U.S. Supreme Court Grant’s Pass ruling, Missouri passed legislation that prohibits unhoused individuals from using “state-owned lands for unauthorized sleeping, camping, or long-term shelters,” which compounds the impact of the shelter bed deficit [33, 34]. In Missouri, the St. Louis metropolitan area has the greatest concentration of unhoused citizens, a population that is disproportionately afflicted by both substance use disorders and severe mental illness.

The opioid use epidemic complicates the state of homelessness in this region. Drug overdose is a leading cause of death among middle aged adults in Missouri. In 2023, there were 1948 deaths from overdoses; over 73% of those fatalities involved opioids, such that 1 out of 47 deaths statewide were attributed to an opioid-involved overdose [35]. In the face of the opioid epidemic, there has simultaneously been a surge in substance use and injection-related infections, including rising rates of Hepatitis C and Human Immunodeficiency Virus (HIV). Missouri has been spotlighted as a region with high rates of HIV transmission and is currently a priority area in the Centers for Disease Control’s Ending the HIV Epidemic campaign [36]. Housing status and the ability engage in traditional healthcare structures complicates the care of living with, and at risk for, HIV [15, 16].

Despite the increasing evidence around many harm reduction practices, such as syringe service programs, safer use supplies and drug testing materials, laws within Missouri have hamstrung harm reduction efforts [37]. Though naloxone and Fentanyl test strips are currently available in Missouri without prescription, the state has not officially approved Xylazine test strips and has resisted legalizing syringe access programs [38, 39]. Thus, options for accessing harm reduction services in the St. Louis region are limited.

Street Medicine St. Louis is a volunteer-based, non-profit organization through which health professionals offer pro bono services to people experiencing unsheltered homelessness. These services take place as “street rounds,” which occur each Saturday morning. These rounds are conducted in the downtown area of St. Louis, which has been home to several large homeless encampments. The founder of the organization has been providing consistent outreach since 2020, with the inclusion of more volunteers beginning in 2022. Given the length and consistency of the outreach, the organization has developed trust within the community. Services rendered include harm reduction support, medical exams, wound care, and the provision of prescription medications, such as medications to treat opioid use disorder, antibiotics, and common over the counter medications. This article is focused on understanding the experiences of unhoused individuals who have received services through Street Medicine St. Louis.

This qualitative study explores how trust influences unhoused individuals' perceptions and utilization of harm reduction materials and education provided by Street Medicine St. Louis. Initially focusing deductively on perceptions and utilization of harm reduction resources, trust emerged inductively as a critical theme. Thus, this manuscript addresses two primary research questions:

1. How does trust between unhoused individuals and outreach providers influence perceptions of harm reduction education and materials?
2. In what ways does trust shape the utilization (or non-utilization) of harm reduction services among unhoused people in St. Louis?

## Methods

This study used qualitative semi-structured interviews to explore how individuals experience outreach medical care, especially harm reduction, in the St. Louis community. This was done primarily by understanding the work of Street Medicine St. Louis as a case example. This study was conducted in the St. Louis region at a low-barrier safe haven shelter, multiple homeless encampments, and on the street with individuals experiencing unsheltered homelessness. Qualitative methods were used to gather personal experiences and stories, allowing participants to share their unique perspectives.

Participants were selected through purposive sampling, and all individuals were 18 and older. As part of the inclusion criteria, all participants had received services from Street Medicine St. Louis. This approach ensured that all individuals had enough experience with these programs to reflect on their experiences and answer the questions of interest. A total of 19 individuals participated in semi-structured qualitative interviews conducted by a member of the street medicine team from April through August of 2023. Two team members were present at each interview. Participants were approached and provided with detailed information about the study’s purpose, and informed consent was conducted prior to any participation in the interviews. To compensate for their time, participants received $15 in the form of a grocery gift card (the current policies at the host institution prevent cash transfers; the price was based on rates for other similar studies in the region). Participation was not tied to whether individuals continued to receive services from Street Medicine St. Louis.

An interview guide (Online Appendix 1) was developed and grouped by assessing harm reduction, education services, and other experiences with street medicine care. Interviews lasted 10–30 min and were conducted in a private or semi-private area to ensure confidentiality. All interviews were recorded and later transcribed in Trint for analysis. Transcripts were de-identified and stored on HIPAA-compliant, password-protected cloud storage hosted through an academic institution. All original recordings were destroyed. This study was reviewed and approved by the Washington University in St. Louis IRB (IRB#: 202302028) and follows the Standards for Reporting Qualitative Research reporting guideline [40].

### Analysis

We analyzed the interview transcripts in the qualitative software program MAXQDA (VERBI Software, 2021) using thematic analysis [41]. The coding team was composed of three of the authors (AG, SM, and DA). We reviewed the interview recordings and transcripts and independently took notes on potential codes and themes related to participants' experiences with Street Medicine St. Louis, their knowledge of harm reduction, and their desires for future harm reduction supplies, educational materials, and events. We met to share our notes and agreed upon a set of initial codes and themes. Next, as a group we analyzed 3 transcripts line-by-line using inductive and deductive coding, continuously revising codes and themes as needed. From there, coder 1 (AG) independently coded 9 transcripts, and coder 2 (DA) independently coded 10 transcripts. Coder 3 (SM) then went through and double-coded 3 transcripts per coder for a total of 6 transcripts or 32% of the sample for reliability. Our group of coders checked in weekly to discuss the process and any questions we encountered while coding. Thematic analysis focused on trust, harm reduction services, and the experiences of unhoused individuals in St. Louis. Themes, as they were developed, were reviewed with the larger research team.

## Results

### Demographics

Nineteen individuals participated in interviews (Table 1). Basic demographics were collected on participants. The participants ranged in age from 27 to 61 (Median: 45; two missing). Ten (52.6%) participants reported their race as Black, and nine (47.4%) reported their race as White. Nine participants (47.4%) reported their gender as female, 9 (47.4%) as male, and one as transgender (5.2%).

**Table 1:** Participant demographics

N = 19	
Age – Median [IQR]	45.0 [38.0 – 49.0]
Race; n (%)	
Black	10 (52.6%)
White	9 (47.4%)
Gender; n (%)	
Female	9 (47.4%)
Male	9 (47.4%)
Transgender	1 (5.2%)

^*^Two values with missing age demographics were excluded from age statistics

#### Theme 1: Navigating trust: relational consistency amid institutional betrayal and structural stigma

##### Subtheme: Building trust through consistent outreach and familiarity

Participants consistently emphasized that their trust in harm reduction and medical services provided by Street Medicine St. Louis depended heavily on regular, predictable outreach and personal familiarity with service providers. When asked whom they preferred to receive services from, participants described consistency as key. Subject 8 explained.

“We know you guys [Street Med STL providers] come down here every Saturday, so like you guys or someone from like [Organization 6], if they would be interested, [Person 1], or like any of them. We know them. We love them. We’re familiar with them, we’re comfortable with them.” (Subject 8)

This consistent presence contrasted sharply with less frequent or impersonal outreach. Subject 8 further clarified:

“Like no offense to the [organization] people that were just here, we appreciate them coming out once a month, but for them to come down here and try to teach us something, they’d be less likely to pay attention.”

Recognition by name was identified as essential to building trust and indicating genuine care rather than performative interactions. Subject 7 emphasized:

“[Organization 8] come out, they greet you, they greet at least four of us by name, so it lets us know they don’t come just to say they’ve been here… you identify us, some of us by name. That’s important to me.”

This sentiment delineates a key difference between performative outreach that is “just to say they’ve been here,” versus relational and affective affirmation built over time. Subject 7 highlighted that Street Medicine St. Louis providers “know us by name, [Organization 8] talk like you guys” which also speaks to intentional communication that was non-pathologizing or stigmatizing. Providers who demonstrated consistent, respectful care created a foundation upon which participants became more receptive to educational messages and interventions.

##### Subtheme: Institutional betrayal and structural stigma as barriers to trust

Trust-building efforts occurred against a background of historical and ongoing institutional betrayal, structural stigma, and repeated systemic failures. Participants’ mistrust of healthcare and emergency systems was directly tied to past negative experiences involving incarceration, medical neglect, or punitive institutional responses, significantly complicating trust-building processes.

Subject 6 described the psychological toll of incarceration resulting from minor infractions:

“Well, you know, it’s like if you miss a court date or something stupid, you know, and then you go to jail, and in [Location 6] in Illinois, they’ll keep you in there. I missed court, and I was in there for 6 months…I felt bad at first, but I began to get bitter. I didn’t feel bad anymore; it just made me want to use more.”

Subject 2 similarly linked significant personal losses to reinitiating substance use, highlighting how systemic neglect exacerbated individual struggles:

“My grandmother… she passed away two years ago. It was her death …and another life event in my life that sent me back out here using... it forced me…I turned to what I knew best: drugs... I ended up being… becoming a shooter. An injector of methamphetamines, also known as crystal or ice. And it, it caused a lot of old things to uh, old habits to resurface. And it, and it’s been going on for two years now.”

Repeated betrayals by healthcare and social service systems amplified internalized stigma, prompting further avoidance of healthcare interactions. Trust, thus, had to be actively rebuilt through consistent, reliable outreach and genuine care by service providers. Subject 1 reflected on the stigma and personal difficulty of relapse:

“I wish I didn’t have to go through these steps to prove to myself… that I could get off the drugs. All I can do is ask for help. I have been to rehab once. I have been to two of them. I have been sober before. 15 years. I relapsed last year. But, I am not mad… the reason is, to show and tell others what I have been through before.”

Subject 6 discussed the stigma associated with obtaining harm reduction supplies from formal settings: “It’s embarrassing, really, to go into the store and buy [needles]. Nobody should be proud. I’m not proud.”

Despite these challenges, some participants actively sought to counteract stigma by modeling harm reduction practices to their peers. Subject 13 encouraged peers to engage in HIV and hepatitis testing by leading by example:

“They were testing [for HIV/Hep C], and people didn’t want to do it. I said come on, let’s go in there and do it, to show an example, and show that there’s nothing wrong with doing it. You got to keep yourself healthy, if you want to live long, you know?... So, once they saw us do it, a lot of other people started doing it. Getting on the van and getting tested and stuff.”

Within a context of deep-seated institutional betrayal and structural stigma, the consistent presence and judgment-free interactions provided by Street Medicine St. Louis emerged as especially critical. Trust-building thus functioned not only as an interpersonal dynamic but as a necessary corrective response to historic and systemic failures, facilitating participants’ meaningful engagement with harm reduction and other essential health services.

##### Subtheme: Trust mitigates fear of carceral consequences

A notable outcome of participants' negative histories with systemic harm and structural stigma was pervasive distrust of emergency services, particularly police. This distrust necessitated internal community management of emergencies, with participants describing a preference for peer-driven harm reduction and overdose reversal due to fears of criminalization and punitive responses.

Subject 8 illustrated how fear of police involvement influenced emergency response behaviors:

“I realized that people tend to walk away when someone has O.D’d [overdosed] because they fear when the cops or ambulance shows up that they’re going to get in trouble… If you call 911 when someone is O.D’ing, you can’t get in trouble… but a lot of people out here don’t trust that.”

Participants consistently emphasized the need for trusted, non-emergency, community-embedded social supports like Street Medicine St. Louis. Outreach providers who built consistent and judgment-free relationships significantly mitigated participants' fears of legal consequences, encouraging more proactive engagement with harm reduction.

For instance, Subject 8 described the complex calculus involved in deciding whether to engage emergency services during an overdose event, noting that formal responses were considered only as a last resort:

“I admit I try to use 911 as a last resort because I know CPR, I know the Narcan… But you have to be breathing in order for it to even enter your system. So, 911 has been called out here, but it’s a last resort… Because a lot of people, even if they had nothing to do with it, prefer the cops not come out here and look at everything.”

The trusted outreach teams significantly reduced these barriers. Participants’ confidence that engaging with street medicine would not lead to negative repercussions such as police involvement facilitated more widespread and consistent harm reduction practices. Nonetheless, distrust of broader emergency systems persisted, influencing decisions around medical care post-overdose. Subject 6 described the challenge of urging medical follow-up after administering naloxone:

“I do [advise further care]. But, most of the time they won’t, so I just advise them to lay down and get a bucket.”

These narratives underscore how trust in non-carceral, harm reduction-focused outreach services meaningfully reduces fear-based avoidance of healthcare among unsheltered persons who use drugs.

#### Theme 2: Rapport as a facilitator of harm reduction knowledge and resource utilization

Participants explicitly connected their trust in outreach providers to their willingness to accept and use harm reduction supplies, such as sterile syringes, fentanyl test strips, and naloxone. When asked whether participants had preferences around the credentials or type of provider they’d prefer to administer STI/HIV and Hepatitis C testing, Subject 12 said, “I don’t think it matters, as long as the person is genuine and what not.” As such, it is clear that credentials are not what confer credibility in this context. Other participants echoed similar sentiments; Subject 11 shared “I am not picky with that because I feel like I can learn something from anybody.”

Interviewers queried whether there are certain people that participants trust more than others, and Subject 12 replied:

“I give everyone the benefit of the doubt. But it only takes one time, and then I am leaving, you know what I am saying? I give everyone the opportunity to prove themselves right, otherwise I question their reliability for it.”

This quote illustrates that even one instance of breaking that trust can rupture relationships between providers and recipients of street medicine. The fragility of trust can be heightened by histories of trauma, scarcity of resources, institutional betrayal, and structural stigma.

Trust not only facilitated initial acceptance but also encouraged active and ongoing use of these resources. Subject 1 was asked how he determines which groups he wants to work with and receive supplies from, versus those he may not trust as much. He gestured to a Street Medicine St. Louis provider, and expressed:

“The doctor that I am sitting with now, he comes out to see us. A very good doctor, I’m glad you come out, thank you. And your assistants, I am glad they come out to see us too. Not many people do that.”

He continued on to mention specific providers by name, indicating the importance of shared recognition and regular contact, and summarized:

“They come out here, and they improve [our circumstances]. Them are good people. They see us. Some of us are struggling, but some of us can make it, if they just have someone to push. I cannot do it on my own. And these are my friends, I love them to the fullest.”

To call providers as friends underscores the importance of relationship-building as a means of promoting harm reduction services. Consistency and mutual respect also enabled participants to voice what they actually wanted and needed. Subject 1 provided this anecdote:

“We ask [redacted name], whenever he comes down here, we might need some extra tents in case another homeless person comes in, and don’t have nowhere to sleep. Now we have a tent to give to that person.”

A key principle of harm reduction is to ‘meet people where they’re at,’ and so if material support like tents are what people ask for, then organizations foster both credibility and reliability through meeting those expressed needs. For instance, when asked whether people in the unhoused community would use a point of care testing site, Subject 8 shared the following:

“As sad as it’s going to say, like you guys [Street Medicine St. Louis], [Organization 8] and stuff like that, if you come up to us, and some of them [people in encampment] have to get water, food, money or whatever…a lot of them I’m afraid would walk right past if there’s no incentive other than good health. Like if you offered a snack or a bottle of water or something, you’re more likely to have a shot. Because we have to choose between getting food and water or going to go get tested, you know what I’m saying? And so, if we can combine the two, it would probably be a lot easier.”

Meeting people’s basic needs that are more urgent is not only integral to the services that street medicine should provide, but also serves as a form of attunement that can promulgate trust and willingness to receive other forms of services. For instance, Subject 7 expressed not only desire for “more info about benzos [benzodiazepines],” but also:

“Those testing strips we get now? I am so glad that you and others bring it out. I’m going to start testing before I do any of this junk. You never know what’s in what and in what amount. No, I can’t do it no more. Benzos? I stopped them a few months ago because they weren’t right or whatever.”

This quote indicates not only Subject 7’s openness to future integration of harm reductive practices, but also how they have already been helpful in her life.

Assessing what will actually be implemented is a necessary step to aligning the distribution and utilization of supplies. Subject 10 was asked whether the bandages and alcohol pads that Street Medicine St. Louis had placed in the harm reduction kits were useful, and he replied: “I’ve never seen it in street drug use period. I never see anybody just wipe off with alcohol like they do in hospitals.” Instead, he suggested the following:

“Another thing I see people never really give… like when you’re living in these tents, it’s a fire hazard, like people need a fire extinguisher in each tent. And I’ve seen so many people when they do the fentanyl or whatever, they nod off and fall in the fire. That guy, his foot caught on fire the other day.”

Integrating such suggestions into supply distribution and service provision is not only tangibly useful for health, but also deepens mutual respect between providers and those receiving their care, with the added benefit of highlighting the relevance of lived experience in addition or even in lieu of formal education and credentialing.

Street Medicine St. Louis providers were able to share critical harm reduction information, not only through distributing resources but also through investing time and interpersonal sharing. Subject 4 was taught how to use test strips by a Street Medicine St. Louis provider, during which time he and the interviewer discussed how an in-person demonstration was much more effective than handing out a pamphlet. In addition, this shared rapport created a conduit for harm reduction service delivery, both in terms of informational (e.g. how to use test strips) and instrumental support (e.g. safer use supplies, wound care). Providers who demonstrated consistent, respectful care created a foundation upon which participants were more receptive to educational messages and interventions.

Other components of the harm reduction kits were lauded as lifesaving. Expressions of gratitude for testing strips was widespread, such as when Subject 2 reflected on a near-death experience:

“I recently died from [a] fentanyl shot that I thought was an ice shot. And, [name redacted] brought me back. And, uh, this right here [holding fentanyl test strip], if I would have had one of these, that day…”

Across the participant responses, naloxone and safer-use supplies are established as extremely important harm reduction practices, with multiple participants providing lived experiences of how they used, or wish they could have used, naloxone to prevent overdoses. Subject 1 provided a detailed account:

“You give them clean needles, clean water. Make sure they have narcan around for the overdose. Make sure someone knows how to use their narcan on that person. Also check if they’re breathing. Make sure you tell them who you are. And, the way I used to do it is, put water on first, on the arm first, on the wound… and then I put it on the back of their neck. But I let them know I am doing it. And if they don’t come out of it then.... then I am going to narcan them. So, once they come out of it, I tell them I’ll hit them with it, how many times I hit them with it. If I don’t have to Narcan them, and they come out of it, I still call 911. Um, we do holler out, ‘Narcan – ASAP,’ someone overdosed. Since I’ve been here, um, I think the last person was [name redacted]. [Turns to look at person outside of interview] I think it was four I had to give you, [name redacted], but I forgot about that, I’m glad I did.”

He also identified how previous experiences with street outreach harm reduction programming taught him about intranasal naloxone, and how to use it, demonstrating the importance of these outreach programs.

Trust extended beyond initial acceptance to influence long-term behaviors and peer-to-peer education and support, with participants reporting higher confidence in distributing and utilizing harm reduction supplies when sourced from trusted outreach providers. Several participants described themselves as informal harm reduction educators and responders, acting on the trust they placed in their outreach team and the tools provided by further disseminating them amongst their peers. This ripple effect across the entire mutual aid network is further explored in the next theme.

#### Theme 3: Collective care and peer-driven harm reduction

Trust within the unhoused community itself was a key facilitator of harm reduction, operating in tandem to or in lieu of formal systems of support. Participants described extensive peer-to-peer care practices, such as overdose reversal, distribution of harm reduction supplies, and health education. These practices reflect a deeply embedded ethic of collective care, with individuals acting as first responders and informal health educators in the absence of formal infrastructure. This sense of shared responsibility was predicated on shared circumstance rather than personal history, including lamenting those they had not met before. Anecdotes of losing loved ones were all the more poignant when intimacy clearly had been cultivated; Subject 6 offered traumatized testimony of having recently lost someone:

“When I found him dead [tearful reply]... Had I had Narcan then, I could have saved him, but I have it now, so… I think that everybody should carry Narcan out here. I Narcaned three people last week.”

Subject 1 articulated how his past experiences learning about harm reduction allowed him to offer life-saving interventions:

“When I came over here, a year ago, I didn't know I was going to have that experience of people overdosing, and when I did, my experiences came out even more. Um, yeah, how can I say this? Two were dead, but I brought them back… I’m glad I was around.”

As a demonstration of their perceived responsibility to the community, participants described carrying and distributing supplies not only for personal use but also to protect others in their encampments. Participant 7 shared, “that’s why I keep clean needles and alcohol pads and hand sanitizer and cotton balls.”

Participant 8 echoed this sentiment, but added how she disseminated the knowledge she gained from harm reduction outreach teams:

“Before I moved out here, almost five years, [Person 2] came out here with [Organization 6], they were out here distributing narcan and other harm reduction supplies and they taught me a little bit about everything, each shot of narcan, and how to do it and everything, they taught us how to do it. I just like to make sure I have supplies on me for other people because I am obviously familiar with what can happen when you’re using the wrong supplies or dirty supplies.”

This quote illustrates that street medicine can serve as the juncture between formal and informal support systems, with a powerful ripple effect that can benefit entire social networks.

Some participants identified specific community roles, such as elders or experienced users, who provided mentorship on safer use, responding to overdoses, or navigating social services. For instance, Subject 2 identified as “the female lead in the community,” and described distributing harm reduction supplies alongside her peers:

“There are other individuals within the community that, uh, hands them out as well, so I am not the only one responsible, and I am actually a member of the board for this community.”

This horizontal mentorship is captured in her strategy:

“Some people... don’t know where to go to get points [needles], and then there’s people like myself that knows where to go to get them…I go get them and then I come back with them and leave them in my tent.”

These efforts were seen as essential to survival. As Subject 1 eloquently reflected:

“Being out here with my fellow friends, is what I call my family. I share. Every day. All day. I try my best to try to keep them focused on [inaudible], no matter what we’re doing out here. Always keep your head up to the sky.”

These practices illustrate that harm reduction was not only something received from outreach teams but was actively practiced and shared within the community outside of professional actors. Collective care functioned both as a survival strategy and a resistance to the isolation and stigma imposed by structural systems.

## Discussion

This study explored how trust influences unhoused individuals’ perceptions and utilization of harm reduction materials and education provided by Street Medicine St. Louis. As a case study of this grassroots street medicine initiative, the findings are uniquely situated within the context of St. Louis, Missouri. Our findings indicate that this outreach program enhances community assets by emphasizing participants' internal and communal strengths. Trust emerged not only as a prerequisite for engagement with services, but as a vital precursor to care, survival, and community health. Trust serves as a key component of how these interactions are colored and how participants engage with outreach teams and programs. These findings are consistent with Boucher et al., who emphasize peer-based, trust-building approaches and the inclusion of user perspectives to enhance the effectiveness and uptake of harm reduction interventions [42]. The five emergent themes demonstrate that trust is both an interpersonal dynamic and a structural response to persistent systems failure.

Participants emphasized that trust was built through regular, familiar, and judgment-free outreach. Recognizing clients by name, showing up consistently, and treating people with respect were seen as critical behaviors that differentiated trusted providers from impersonal or punitive institutions. These findings align with literature on trauma-informed care and street-based interventions, which identify relational consistency and emotional safety as foundational to successful engagement with individuals who have experienced structural violence and stigma [13, 25, 43, 44]. In Missouri, where formal services are often inaccessible and medical mistrust is exacerbated by racial disparities in healthcare access, this consistency is particularly crucial.

Second, trust enabled participants to accept and use harm reduction strategies. Participants described using naloxone and fentanyl test strips themselves, intervening during overdoses, and mentoring others. These acts of care represent a community-based approach to harm reduction, where informal peer networks act as vital sources of support acting in tandem to structural constraints, reflecting Rhodes et al.'s (2005) concept of the “social production of risk.” Limited access to syringe exchange programs and test strips, in addition to punitive drug laws, increase harm and reduce options for care, making trusted, informal systems of support all the more essential.

Third, participants described a pervasive fear of punitive surveillance and criminalization, especially when seeking emergency medical assistance. Outreach providers who operated independently of police and provided harm reduction and medical services without judgment or surveillance mitigated this fear. Missouri’s legislative environment—such as the criminalization of sleeping on public lands and resistance to Good Samaritan law expansion—compounds this anxiety and contributes to systemic avoidance of formal institutions. In this context, trust in providers served as a necessary condition for harm reduction engagement, and peer support networks attenuate service gaps.

Fourth, participants’ lives were shaped by experiences of structural stigma—discriminatory policies and institutional practices that limit opportunities for housing, health care, and safety—as well as social stigma, the enacted prejudice and judgment from others, and self-stigma, or the internalization of those beliefs. Experiences of incarceration, medical neglect, and rejection from social services were common, and many participants shared how these encounters led to withdrawal from public systems altogether. Trust had to be rebuilt through consistent, person-centered care. Street Medicine St. Louis achieved this by fostering relational and non-hierarchical forms of engagement, which stand in stark contrast to the coercive or harmful structures participants had previously encountered.

Finally, this study also underscores the importance of community-based harm reduction, in which people who use drugs serve as first responders, educators, and caregivers. Participants described naloxone administration, supply sharing, and overdose response as routine features of camp life. These networks operate as a parallel infrastructure of care, especially in environments like St. Louis where formal harm reduction services are underfunded or absent. These findings align with Switzer et al., emphasizing the importance of innovative, community-oriented strategies for harm reduction [45]. As in other cities, collective care is a defining feature of survival strategies among unhoused communities and must be recognized, supported, and resourced by public health agencies.

### Implications

This case study contributes to a growing body of literature that demonstrates how trust is contextually produced and relationally sustained, especially in hostile or neglectful policy environments. Programs like Street Medicine St. Louis are uniquely effective because they operate at the intersection of healthcare and community solidarity. Their success offers a model for other jurisdictions navigating similar barriers.

In St. Louis, where structural racism shapes housing access, healthcare outcomes, and criminal justice interactions, trust-building efforts cannot be separated from broader equity goals. Harm reduction programs must address structural stigma by advocating for policy reforms; they must address social stigma by training providers in cultural humility and anti-racist practice; and they must address self-stigma by affirming dignity, agency, and peer leadership in every encounter.

Policy reforms in Missouri are urgently needed to support harm reduction infrastructure. This includes legalizing syringe service programs, expanding protections for overdose responders, approving safer supply tools like Fentanyl and Xylazine test strips, and ending the criminalization of homelessness. Until then, low-barrier outreach and peer-led education remain the most viable and humane interventions available.

### Limitations

This study has several limitations. First, although guided by principles of qualitative inquiry, the original purpose of the interviews was program evaluation, which limited both the depth of probing and the rigor of data collection. Interviews were brief, and data saturation was not systematically assessed. Second, all participants had received services from Street Medicine St. Louis, potentially introducing selection bias and limiting generalizability. Third, the research team included program affiliates, which may have influenced participant responses due to social desirability bias.

Future studies should incorporate longer, in-depth interviews and include individuals not currently connected to outreach services, in order to explore divergent perspectives and unmet needs. More participatory or community-based approaches could also enhance validity and ensure that analysis is grounded in the lived realities of the population studied.

## Conclusion

This study demonstrates that trust is both a barrier and a bridge to harm reduction and street medicine services among unhoused people who use drugs. Trust, when established through consistent and non-judgmental outreach, enables the uptake of harm reduction education and materials, facilitates community-based overdose response, and supports peer leadership in health promotion. Conversely, mistrust—shaped by histories of incarceration, structural stigma, and institutional neglect—can impede life-saving care.

The relevance of these findings lies in their potential to inform the design and delivery of harm reduction programs. As jurisdictions grapple with intersecting crises of housing instability and overdose, programs that foreground trust and partnership—especially with peer leaders—are more likely to be accepted and sustained. Trust is not ancillary to care; it is a core component of effective harm reduction.

To support the health and dignity of people who use drugs and experience homelessness, public health and social service systems must adopt relational, community-centered approaches that honor lived experience, address structural inequities, and build the kind of trust that enables survival.

## Supplementary Information

Below is the link to the electronic supplementary material.

Supplementary Material 1

## Data Availability

No datasets were generated or analysed during the current study.

## References

[1] Marmot M. Social determinants of health inequalities. Lancet. 2005;365(9464):1099–104. 15781105 10.1016/S0140-6736(05)71146-6

[2] Sousa Td, Andrichik A, Prestera E, Rush K, Tano C, Wheeler M. The 2023 annual homelessness assessment report (AHAR) to Congress. Te U.S. Department of Housing and Urban Development: OFFICE OF COMMUNITY PLANNING AND DEVELOPMENT; 2023.

[3] Sousa Td, Henry M. The 2024 Annual Homelessness Assessment Report (AHAR) to Congress. 2024.

[4] Office UGA. Homelessness: better HUD oversight of data collection could improve estimates of homeless population. 2020.

[5] Liu M, Sandhu S, Feldman BJ, Munson DG. Health care beyond clinic walls - sustaining and scaling up street medicine. N Engl J Med. 2024;390(23):2136–9. 38884328 10.1056/NEJMp2314560

[6] Hossain MM, Sultana A, Tasnim S, Fan Q, Ma P, McKyer ELJ, et al. Prevalence of mental disorders among people who are homeless: an umbrella review. Int J Soc Psychiatry. 2020;66(6):528–41. 32460590 10.1177/0020764020924689

[7] Beijer U, Wolf A, Fazel S. Prevalence of tuberculosis, hepatitis C virus, and HIV in homeless people: a systematic review and meta-analysis. Lancet Infect Dis. 2012;12(11):859–70. 22914343 10.1016/S1473-3099(12)70177-9 PMC3494003

[8] Taylor SN, Munson D. Health care of people experiencing homelessness: part II. NEJM Evid. 2023;2(9):e2300175. 10.1056/EVIDra2300175 38320194

[9] Richards J, Kuhn R. Unsheltered homelessness and health: a literature review. AJPM Focus. 2023;2(1):100043. 37789936 10.1016/j.focus.2022.100043 PMC10546518

[10] Narayan A, Shah N, Hochman M. Beyond brick and mortar: the rise of street medicine. J Gen Intern Med. 2024. 10.1007/s11606-024-08880-x. 38937362 10.1007/s11606-024-08880-x PMC11534948

[11] Kaufman RA, Mallick M, Louis JT, Williams M, Oriol N. The role of Street Medicine and mobile clinics for persons experiencing homelessness: a scoping review. Int J Environ Res Public Health. 2024. 10.3390/ijerph21060760. 38929006 10.3390/ijerph21060760 PMC11204218

[12] Street Medicine: Medical outreach for unsheltered people [press release].

[13] Frankeberger J, Gagnon K, Withers J, Hawk M. Harm reduction principles in a street medicine program: a qualitative study. Cult Med Psychiatry. 2023;47(4):1005–21. 36229766 10.1007/s11013-022-09807-z PMC9560723

[14] Institute SM. The street medicine institute story [Available from: https://www.streetmedicine.org/our-story.

[15] Armstrong W, Evans, T.B., Tookes, H. III. To end the HIV epidemic, we need to reach unsheltered homeless populations. Healthaffairs.org; 2023 [Available from: https://www.healthaffairs.org/content/forefront/ending-hiv-epidemic-require-embracing-alternative-ways-reach-unsheltered-homeless.

[16] Killelea A, Armstrong W, Barocas J, Bergholz D, Corbin-Gutierrez E, Dombrowski J, et al. A systems level approach to innovative HIV care and treatment models in the United States: street medicine and differentiated service delivery HIV medicine association; 2023.

[17] Feldman BJ. Streetmedicine.org2023. Available from: https://www.streetmedicine.org/index.php?option=com_dailyplanetblog&view=entry&year=2023&month=06&day=27&id=33:new-pos-code-for-street-medicine-from-cms.

[18] Feldman BJ, Feldman CT, Kogan AC, Saluja S, Cousineau M. The California street medicine landscape survey and report. 2023.

[19] Homelessness. USICo. Core elements of effective street outreach to people experiencing homelessness. 2019.

[20] Council NHCftH. Street medicine and outreach: bringing care to people where they are. healing hands; 2022.

[21] Coalition NHR. Principles of harm reduction [Available from: https://harmreduction.org/about-us/principles-of-harm-reduction/.

[22] Rich KM, Solomon DA. Medical Complications of Injection Drug Use — Part I. NEJM Evidence. 2023;2(2). 10.1056/EVIDra2200292 38320040

[23] Rich KM, Solomon DA. Medical complications of injection drug use—Part II. N Engl J Med Evid. 2023. 10.1056/EVIDra2300019. 10.1056/EVIDra2300019 38320028

[24] Sue KL, Fiellin DA. Bringing harm reduction into health policy—combating the overdose crisis. NEJM Evid. 2021;84(19). 10.1056/NEJMp2103274 33983692

[25] Chan CA, Canver B, McNeil R, Sue KL. Harm reduction in health care settings. Med Clin North Am. 2022;106(1):201–17. 34823731 10.1016/j.mcna.2021.09.002

[26] Regenstein MP, Mead KHP, Trott JM, Seyoum SM, Baños JM, Van Bronkhorst H, et al. The public health response to COVID-19 in the St. Louis Region of Missouri. 2022.

[27] Minnesota Uo. SHADAC Analysis of Per person state public health funding. State Health Compare.

[28] Government SLC. FY2025 Annual Operating Plan: Health and Hospitals: St. Louis City Government; 2024 [Available from: https://www.stlouis-mo.gov/government/departments/budget/documents/upload/FY2025-AOP-Health-and-Hospitals.pdf.

[29] Government SLC. Homelessness: the rate at which residents receive emergency housing from service providers funded by the City of St. Louis Department of Human Services [Available from: https://www.stlouis-mo.gov/government/departments/mayor/initiatives/resilience/equity/opportunity/health-safety/homelessness.cfm.

[30] Purnell J, Camberos G, Fields R. For the sake of all: a report on the health and well-being of African Americans in St. Louis and Why It Matters for Everyone Washington University in St. Louis and Saint Louis University; 2014.

[31] Development USDoHaU. HUD 2023 Continuum of care homeless assistance programs homeless populations and subpopulations: Missouri. 2023 November 20, 2023.

[32] America HSDHtNo. Missouri homeless shelters. 2024.

[33] Keen R, WInkleby M. Criminalizing homelessness—the grants pass, oregon, supreme court case. N Engl J Med. 2024. 10.1056/NEJMp2405434. 38810183 10.1056/NEJMp2405434

[34] Chapter 67 Political Subdivisions, Miscellaneous Powers: Homelessness, use of state funds, when — state-owned lands not to be used for unauthorized sleeping, camping, or shelters — no state funding, when — rules, (2023).

[35] Services MDoHaS. Drug overdose dashboard - fatal overdoses. 2023.

[36] CDC. Ending the HIV epidemic in the US jurisdictions and plans: Local EHE Plans [Available from: https://www.cdc.gov/ehe/php/jurisdictions-plans/index.html.

[37] (SAMHSA) SAaMHSA. Harm reduction framework. Substance Abuse and Mental HEALTH Services Administration; 2023.

[38] Nickelson P. Xylazine-involved fatal drug overdoses in Missouri 2019–2022. Missouri Department of Health and Senior Services; 2023 May 11, 2023.

[39] Health MFf. Harm reduction: a strategy to reduce the toll of substance use in Missouri. 2021.

[40] O’Brien BC, Harris IB, Beckman TJ, Reed DA, Cook DA. Standards for reporting qualitative research: a synthesis of recommendations. Acad Med. 2014;89(9):1245–51. 24979285 10.1097/ACM.0000000000000388

[41] Clarke V, Braun V. Thematic analysis. J Posit Psychol. 2016;12(3):297–8.

[42] Boucher LM, Marshall Z, Martin A, Larose-Hebert K, Flynn JV, Lalonde C, et al. Expanding conceptualizations of harm reduction: results from a qualitative community-based participatory research study with people who inject drugs. Harm Reduct J. 2017;14(1):18. 28494774 10.1186/s12954-017-0145-2 PMC5427533

[43] Claborn K, Samora J, McCormick K, Whittfield Q, Courtois F, Lozada K, et al. “We do it ourselves”: strengths and opportunities for improving the practice of harm reduction. Harm Reduct J. 2023;20(1):70. 37296459 10.1186/s12954-023-00809-7 PMC10250854

[44] Medellin T, Moczygemba LR, Thurman W. A qualitative study to describe the nature and scope of street medicine programs in the United States. Int J Environ Res Public Health. 2024. 10.3390/ijerph21121623. 39767464 10.3390/ijerph21121623 PMC11675444

[45] Switzer S, Guta A, de Prinse K, Chan Carusone S, Strike C. Visualizing harm reduction: methodological and ethical considerations. Soc Sci Med. 2015;133:77–84. 25841098 10.1016/j.socscimed.2015.03.040

