# Supplementary material for: Understanding the role of street medicine in harm reduction: a case study of Street Medicine St. Louis
Source: Harm Reduct J. 2025 Oct 6;22:161. doi: 10.1186/s12954-025-01313-w (PMC12502220; doi:10.1186/s12954-025-01313-w)
Supplement: Supplementary file 1 — Supplementary Material 1 [file 12954_2025_1313_MOESM1_ESM.docx]

**Appendix 1. Interview Guide**

Overview/Introduction

Thank you for taking the time to talk to me today. We are interested in a little bit more about how you get information about drug use, and where you might get resources for safe drug use and wound care. We are hoping to get a better understanding of your experiences and perceptions regarding the available things like education, resources you get, and testing that you might have been asked to use.

First, I am going to ask about education materials. We are going to record this so that I can make sure I understand what you have said later. Your name will not be attached to any of this. If at any point you want me to take something out of the recording, let me know. Also, you can let me know if you want to skip a question. I might ask you a question that seems obvious, but that is because I want to make sure I am correctly understanding what you say.

1. Education Materials
   1. What do you currently understand about harm reduction? What does harm reduction mean to you?
   2. Has anyone ever talked to you about how to use drugs more safely?
   3. Do you want people to talk to you more about things like: safe drug use practices? Resources you can use? Wound care?
      1. What would you like to learn? How would you like people to give you the information? In what format (paper, someone talking to you, etc)?
   4. When you need to learn something about ___, where do you go for information? Where would you like to receive education?
   5. Are there people you feel like you trust more than others? Are there certain people that you would want to talk to?
   6. What else would you like to learn?
   7. Are the educational materials easy or difficult to understand?
2. Harm Reduction Kit Questions
   1. Could you share with me a little about how you use your drugs? Take me through the step by step?
   2. What materials would help you use drugs in a safer way? Is there something that you feel like you could use but don’t have regular access to?
   3. How do you currently receive supplies? What is the best way to receive harm-reduction supplies?
   4. What materials in our harm-reduction kits do you find to be the most useful?
   5. What materials in our harm-reduction kits do you find to be the least useful?
   6. Do you keep Narcan on you? Do you know of people around you who have Narcan?
   7. How often do you test your drugs for fentanyl?
3. Point of Contact Testing Questions
   1. Do you know your HIV status? When was your last test?
   2. Do you know your Hep C status? When was your last test?
   3. What has been your experience with testing in the past?
   4. Would you like to receive testing for HIV and Hep C from us?
   5. How do you feel about HIV and Hep C tests being given here where you live?
   6. How do you feel about HIV and Hep C tests being performed by someone other than a doctor? Who would you want to talk to about testing?
